# Deciphering the Structure and Genetic Basis of Adaptive Mechanism of Soil Microbial Communities in a Manganese Electrolysis Plant

**DOI:** 10.3390/microorganisms14010015

**Published:** 2025-12-20

**Authors:** Yong Wang, Song Liu, Ziyi Zheng, Jun Ma, Yuan Xiang, Lanyan Wu, Chunlian Ding, Yan Shi

**Affiliations:** 1 College of Materials and Chemical Engineering, Tongren University, Tongren 554300, China; 2 College of Environment and Ecology, Chongqing University, Chongqing 400044, China; 3 College of Materials Science and Engineering, Chongqing University, Chongqing 400044, China; 4 School of Metallurgy and Environment, Central South University, Changsha 410083, China

**Keywords:** manganese contamination, metagenomics, soil microorganisms, environmental drivers

## Abstract

The development of China’s manganese (Mn) industries has caused severe water and soil pollution, threatening ecological and human health. Microbes are usually regarded as an important indicator of environmental pollution assessment. However, the current understanding of microbial community characteristics and their formation mechanisms in Mn production areas remains limited. In order to address this, soil properties and microbial structural characteristics across different functional zones in a typical Mn electrolysis plant in China’s “Manganese Triangle” were investigated via metagenomic sequencing. Results showed soil Mn levels significantly exceeded background values, indicating high environmental risk. *Acidobacteria* and *Proteobacteria* were dominant phyla. Microbial abundance was lowest in the adjacent natural reservoir, whereas diversity was highest in the sewage treatment plant. Correlation analyses identified Mn, nitrate nitrogen, ammonium nitrogen, pH, and moisture as key environmental drivers, with Mn being the primary one. Metagenomic analysis revealed abundant Mn resistance genes, enabling microbial survival under high Mn stress. This study demonstrated that excessive Mn exposure enriched Mn-resistant genes, thereby shaping unique microbial communities dominated by Mn-resistant bacteria. These findings clarified the structural characteristics and adaptive mechanisms of soil microbial communities in Mn-contaminated areas, providing a theoretical basis for ecological risk management and bioremediation.

## 1. Introduction

Manganese (Mn) and its compounds are key materials for industries such as metallurgy, the chemical industry, steel, and medicine. As these industries have rapidly developed, the demand for Mn has been increasing [1]. These processes of ore mining, beneficiation, and electrolysis for Mn production can lead to serious environmental pollution, posing threats to the health of flora, fauna, and humans [2,3]. Especially, Mn electrolysis processes can generate wastewater and slag containing complex pollutants, including heavy metals, Mn, and ammonia nitrogen [4]. Owing to rainwater erosion and surface runoff, the surrounding soil faces the incredible risk of high Mn pollution. Songtao County, located in Tongren City in Guizhou Province, is one of the primary regions for Mn resources in China. The surrounding soil is at a potentially high risk of Mn and heavy metal contamination. Recent research has revealed excessive levels of soil heavy metals (e.g., Mn, 4042.15–13,635.14 mg/kg; Cd, 0.70–1.57 mg/kg; Pb, 35.18–38.47 mg/kg) in a manganese mine area in Tongren, Guizhou [5], which pose incalculable risks to local ecosystems and human health. Therefore, the impact of soil Mn and heavy metal pollution on the ecosystem environment in the manganese mine areas of Tongren, Guizhou, should not be underestimated.

Microorganisms are the critical part of the soil ecosystem and play key roles in element circulation and energy transport [6]. Microbial communities are dynamic and sensitive to external environmental change, which in turn alters their structure [7]. Therefore, soil microorganisms are an important indicator of the soil environment. Several studies have shown that Mn pollution in mining areas can change the soil microbial community structure and affect their biological metabolic capacity [8,9]. Xiang et al. found that the soil microbial community around the mining area was affected by heavy metals and that species complexity was positively correlated with distance from the manganese mine area [10]. An investigation of the structure and abundance of the moss rhizobial communities in manganese mining areas of South China found that the diversity and community structure of moss rhizobia were significantly related to the levels of heavy metal contamination, and bacteria with strong tolerance to heavy metals were the dominant species in mine soils [5]. In addition, other environmental factors in the manganese mine area can affect the microbial community and composition. Another study examined the impact of restoration with moss cover on the soil microbial community in a manganese slag heap area found the dominant families are *Bryaceae* (50%) and *Pottiaceae* (25%) [11]. And the composition of soil bacteria at the phylum level was consistent across different successional stages, while the abundance of each bacterial group varied with the succession development of moss. These previous studies have explored the impact of Mn pollution on microbial communities from multiple perspectives, but the underlying mechanisms for the changes in microbial community structure caused by metal pollution remain unclear.

Since genes are the fundamental determinants of microbial taxonomy, function, and environmental adaptive traits, metagenomic profiling of soil microbes can effectively reveal the underlying adaptive mechanisms of microbial communities in Mn-contaminated sites. Here, a metagenome-based integrated approach was conducted to investigate the impact of a typical manganese electrolysis enterprise in Bijiang District, Tongren City, China, on soil ecosystems. The spatial distribution of Mn and key physicochemical properties across different functional zones was characterized. Using shotgun metagenomic sequencing, we profiled the diversity and composition of soil microbial communities. The correlation between environmental factors and microbial profiles was analyzed to identify the primary driver shaping microbial community structure. Specifically, we quantified the abundance and distribution of Mn-related functional genes to elucidate the adaptive mechanism enabling microbial survival in this contaminated environment. Our findings are expected to provide a theoretical basis for developing risk management and bioremediation strategies for manganese-contaminated soils.

## 2. Materials and Methods

### 2.1. Sample Collection and Processing

**Figure 1:**
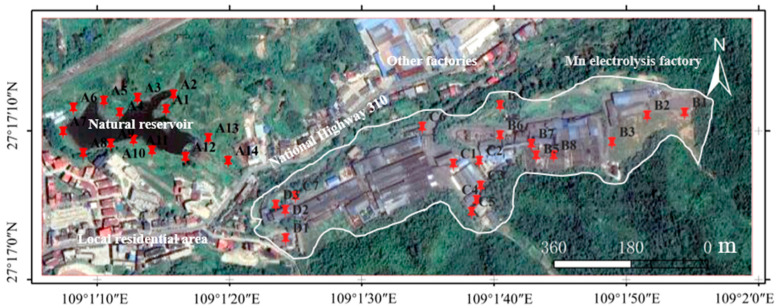
Location of the sampling sites from a manganese electrolysis factory and its surrounding environment in the Bijiang District, Tongren City, Guizhou Province (Red pins represent the actual sampling points. Notably, the Mn electrolysis factory is located at a slightly higher elevation than its surroundings).

Soil samples were obtained from an electrolytic manganese enterprise and its surrounding environment in the Bijiang District, Tongren City, Guizhou Province, China. The area features hilly terrain, and dense green vegetation covers the undisturbed hills. The boundary, outlined in white in Figure 1, encompasses the primary production infrastructure—a cluster of long, linear buildings and disturbed areas centrally located between coordinates 109°1′20″ E and 109°2′0″ E. The manganese electrolysis factory is situated on elevated ground, while local residential areas, other industrial facilities, and natural reservoirs are in topographically lower areas. Based on the different functional divisions of the factory, surface soil (≤30 cm) was sampled from the adjacent natural reservoir (Zone A, samples A1–A14), production and slag heap zones (Zone B, samples B1–B7), office zones (Zone C, samples C1–C8), and sewage treatment units (Zone D, samples D1–D3). At the same time, local background soil samples were collected as controls [12]. The random distribution method was used for sample collection. At each site, samples were collected four times in a Z-shaped position and mixed evenly to form one sample. All samples were sealed in plastic bags for storage and transport. Part of the soil samples was immediately placed in a −80 °C freezer for metagenomic sequencing and analysis. The residual soil was naturally air-dried, ground, and sieved to determine its physicochemical properties and Mn content. All soil samples were stored at 4 °C until further use.

### 2.2. Determination of Soil Physicochemical Properties

The environmental indicators of all the samples were measured, including physicochemical properties and Mn content, according to previous studies [13]. The soil samples were dried at 105 ± 2 °C to a constant weight to estimate the soil moisture. The soil pH was determined by using a glass electrode in a water–soil ratio (*w*/*w*) of 2.5:1. Soil organic matter (TOC) was determined by the combustion oxidation-titration method (HJ 658-2013) [14]. Briefly, air-dried soil is heated to oxidize organic carbon to CO_2_. The CO_2_ is trapped by an excess Ba(OH)_2_ solution, and the residual Ba(OH)_2_ is titrated with oxalic acid to determine the CO_2_ yield for calculating organic carbon content. Soil nitrogen, including nitrate nitrogen (NO_3_^−^-N) and ammonium nitrogen (NH_4_^+^-N) was extracted with potassium chloride solution, then determined by spectrophotometric methods (HJ 634-2012) [15]. NO_3_^−^-N is quantified spectrophotometrically at 543 nm via the red azo dye formed by the diazotization and coupling reaction with Griess reagents. NH_4_^+^-N is measured at 630 nm based on the formation of a blue indophenol dye with phenol and hypochlorite in alkaline medium. Soil-soluble sulfate (SO_4_^2−^) was quantified by ion chromatography. Soil Mn was determined by inductively coupled plasma mass spectrometry (ICP-MS NexION 300X, PerkinElmerE, Waltham, MA, USA) after digestion with HNO_3_-HClO_4_-HF triacid. All analyses were performed in triplicate.

### 2.3. Soil DNA Extraction and Metagenomic Sequencing

Soil DNA was extracted using a Soil DNA Extraction Kit (Qiagen, Venlo, The Netherlands). Then, the qualified genomic DNA was randomly fragmented to an average size of approximately 300–400 bp using ultrasonication. After that, the sequencing library was established by the following standard steps: end-polished, A-tailed, full-length ligated, and purified PCR products. The library was sequenced using Illumina PE150, and raw data were generated. The raw data were filtered using fastp software (fastp v0.23.4) to obtain clean data. The clean data were then de novo assembled into contigs using MEGAHIT (Assembly Software, V1.04-beta) [16], and the assembly results were evaluated using QUAST software (QUAST 5.3.0) [17]. All sequences can be found under the NCBI BioProject PRJNA1289730. The 32 metagenomes and the metadata can be found under the accession numbers SAMN49894097-SAMN49894128. Protein-coding genes were predicted on the contigs using MetaGeneMark (https://genemark.bme.gatech.edu/genemark/meta_gmhmmp.cgi, accessed on 27 June 2025, Version 3.56). These genes were then taxonomically classified by alignment to public databases (e.g., Non-Redundant Protein Database, Gene Ontology), and functionally annotated against databases like Kyoto Encyclopedia of Genes and Genomes (KEGG) and eggNOG. The number and abundance of genes at each classification level in each sample were identified based on the BLAST hits (BLAST+ 2.17.0) against these databases.

### 2.4. Metagenomic Data Analysis and Taxonomic Profiling

Taxonomic classification of the metagenomic sequences was performed to profile the microbial community composition across all domains of life. Classification was performed by translating the reads into amino acid sequences and searching them against the NCBI non-redundant protein database (Nr database) containing reference sequences from bacteria, archaea, viruses, and eukaryota. The relative abundance of taxa at different taxonomic ranks (from phylum to genus) was calculated based on the number of assigned reads. For downstream ecological analyses (e.g., α-diversity, β-diversity, and correlation with environmental factors), the focus was placed on the bacterial community due to its dominance (>95% of assigned sequences) and its central role in soil metal biogeochemistry in the studied environment. The relationships between environmental factors and microbial community structure were illustrated using Canonical Correlation Analysis (CCA) and Spearman correlation analysis (IBM SPSS Statistics 25.0) and visualized using Origin 2023. Furthermore, genes related to Mn resistance were identified from the gene annotation results of soil metagenome, and their abundance across different functional zones was analyzed. To identify the putative microbial hosts of manganese resistance, a Spearman rank correlation analysis was performed. The relative abundance of each manganese resistance gene was correlated with the relative abundance of the dominant microbial phyla across all samples. Correlations with a *p* < 0.05 were considered statistically significant.

### 2.5. Statistical Analysis

Statistical analyses were performed using SPSS version 25.0. The Shapiro–Wilk method was used to test the normality of each variable. Continuous variables conforming to the normal distribution were shown as the mean ± standard deviation ($\bar{x}$ ± s), and the *t*-test for two independent samples was used for comparison between two groups, while one-way analysis of variance (ANOVA) was used for comparisons among multiple independent groups. Continuous variables conforming to a non-normal distribution are shown as medians and quartiles. The Mann–Whitney U test was used for comparisons between two groups, and the Kruskal–Wallis H test was used for comparisons among multiple groups. The test level was set to α = 0.05.

## 3. Results

### 3.1. Soil Physicochemical Properties

**Figure 2:**
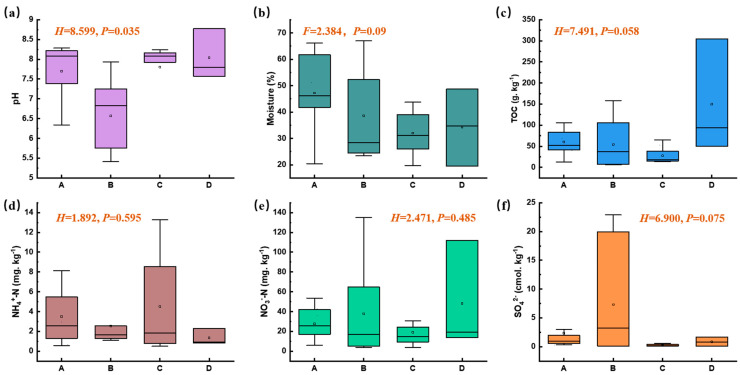
Physico-chemical characteristics of soil samples across different functional zones (Moisture content, ANOVA test; other variables, nonparametric Kruskal–Wallis test, *p* < 0.05; A, B, C, and D represent zones A, B, C, and D).

The surface soil in this plant area exhibited a wide pH range, from acidic to alkaline (5.41 to 8.77), and there were significant differences in pH values across different functional zones (nonparametric Kruskal–Wallis test, *p* < 0.05, Figure 2a). Compared with other zones, the pH level in Zone B was relatively lower. The moisture content ranged from 19.51% to 66.97%, and there were no significant differences among different functional zones (*p* > 0.05, Figure 2b), with the moisture content in the adjacent natural reservoir zone (Zone A) being slightly higher than that in other zones. There were no significant differences in the TOC, NO_3_^−^-N, NH_4_^+^-N, and SO_4_^2−^ contents among different functional zones (*p* > 0.05, Figure 2c–f). Previous studies have suggested that ammonia nitrogen is one of the main pollutants in the manganese electrolysis process [18], whereas soil NO_3_^−^-N and NH_4_^+^-N in this electrolytic manganese plant were at normal levels. This indicates that nitrogen pollution has been effectively controlled since it was incorporated into the total control indicators during China’s 12th Five-Year Plan period.

**Figure 3:**
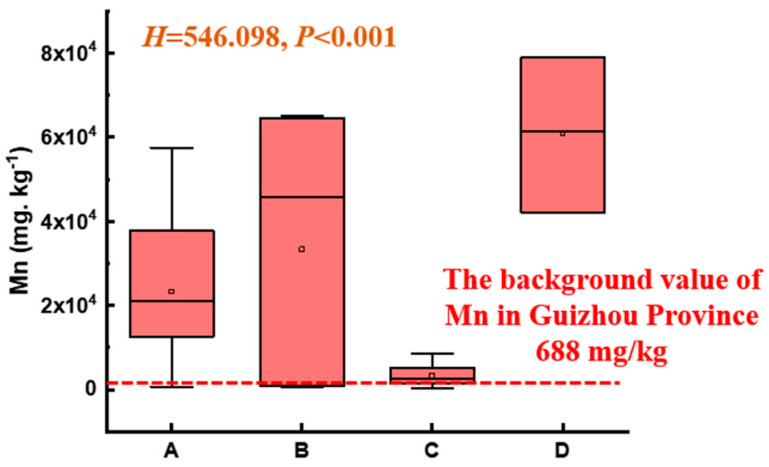
Mn content of soil across different functional zones (nonparametric Kruskal–Wallis test, *p* < 0.05; A, B, C, and D represent Zone A, B, C, and D).

As shown in Figure 3, the soil Mn level in the electrolytic manganese plant significantly exceeded the background value of Mn in the Guizhou Province (668 mg/kg) [19], with significant differences among different functional zones (*p* < 0.001). Specifically, the ranking of Mn levels was Zone D > Zone B > Zone A > Zone C. The maximum Mn content in the soil surrounding the natural reservoir (Zone A) was 50 times the background value. The soil Mn in the production and slag heap zones (Zone B) and sewage treatment units (Zone D) reached 82 and 100 times the background value, respectively. The Mn content in the office zones (Zone C) was relatively low, yet individual points exceeded the normal range by a large margin.

### 3.2. Characteristics of Microbial Community Structure

#### 3.2.1. Microbial Community Diversity

**Figure 4:**
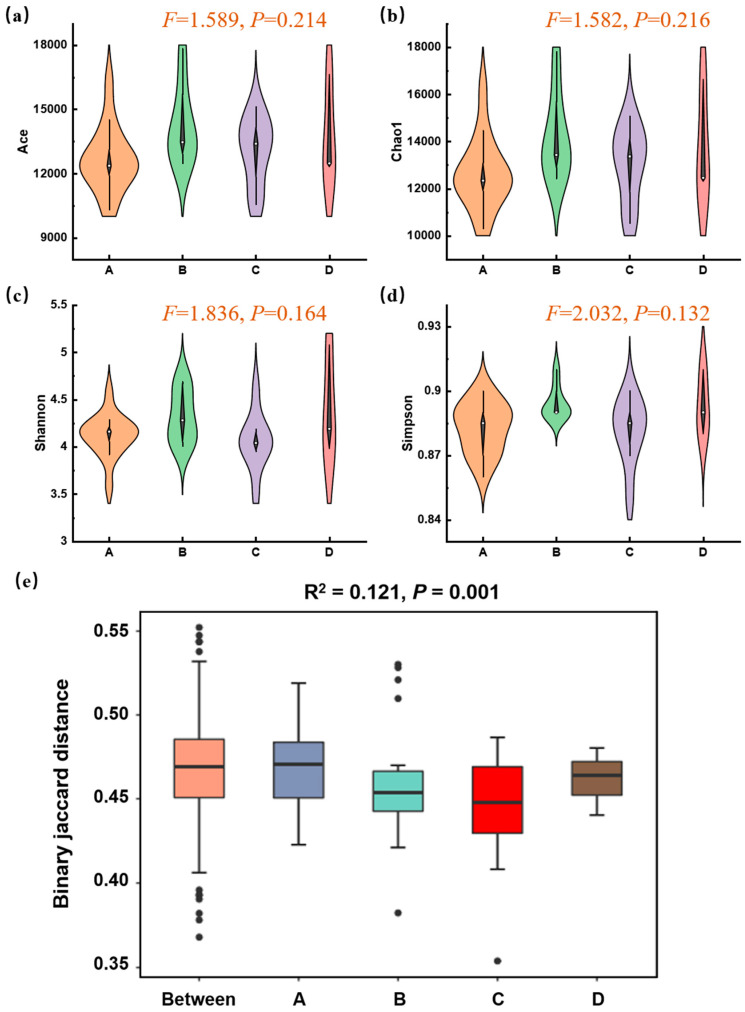
Microbial α- and β-diversity across different zones of Mn electrolysis plant. (**a**–**d**) Violin plots of richness (Chao1, ACE) and diversity/evenness (Shannon, Simpson) indices within zones. (One-way ANOVA, *p* < 0.05); (**e**) Boxplots based on binary Jaccard distances show the dissimilarity within each zone (A, B, C, D) and between randomly assigned groups (Between) (PERMANOVA, *p* = 0.001; A, B, C, and D represent Zone A, B, C, and D).

Based on metagenomic data assembly and filtering, an average of 569,449 contig sequences per sample was obtained, indicating that it was feasible to effectively elucidate the presence of most microorganisms within soil samples. The α-diversity of the microbial communities was assessed based on metagenomic annotation results. ACE and Chao1 indices serve as metrics for assessing microbial richness, whereas Shannon and Simpson indices are used to evaluate microbial community diversity. As shown in Figure 4a–d, the α-diversity indices across the four functional zones showed some variations but no statistically significant differences (*p* > 0.05). This indicated that while local species richness and evenness varied, the overall within-sample diversity was not simply reduced by the Mn contamination gradient. Therefore, to decipher the impact of Mn, we focused on the compositional differences between communities (β-diversity). A PERMANOVA test based on Jaccard distance revealed a highly significant effect of the functional zone on the overall microbial community structure (*p* = 0.001, Figure 4e). This suggested that the communities in different zones were compositionally distinct from one another, and this difference was unlikely to have occurred by chance. The “Between” group, representing a null model of random dissimilarity, was significantly lower than the observed inter-zone differences, confirming that a strong environmental filter, such as Mn contamination, was actively structuring the communities.

#### 3.2.2. Microbial Community Structure

**Figure 5:**
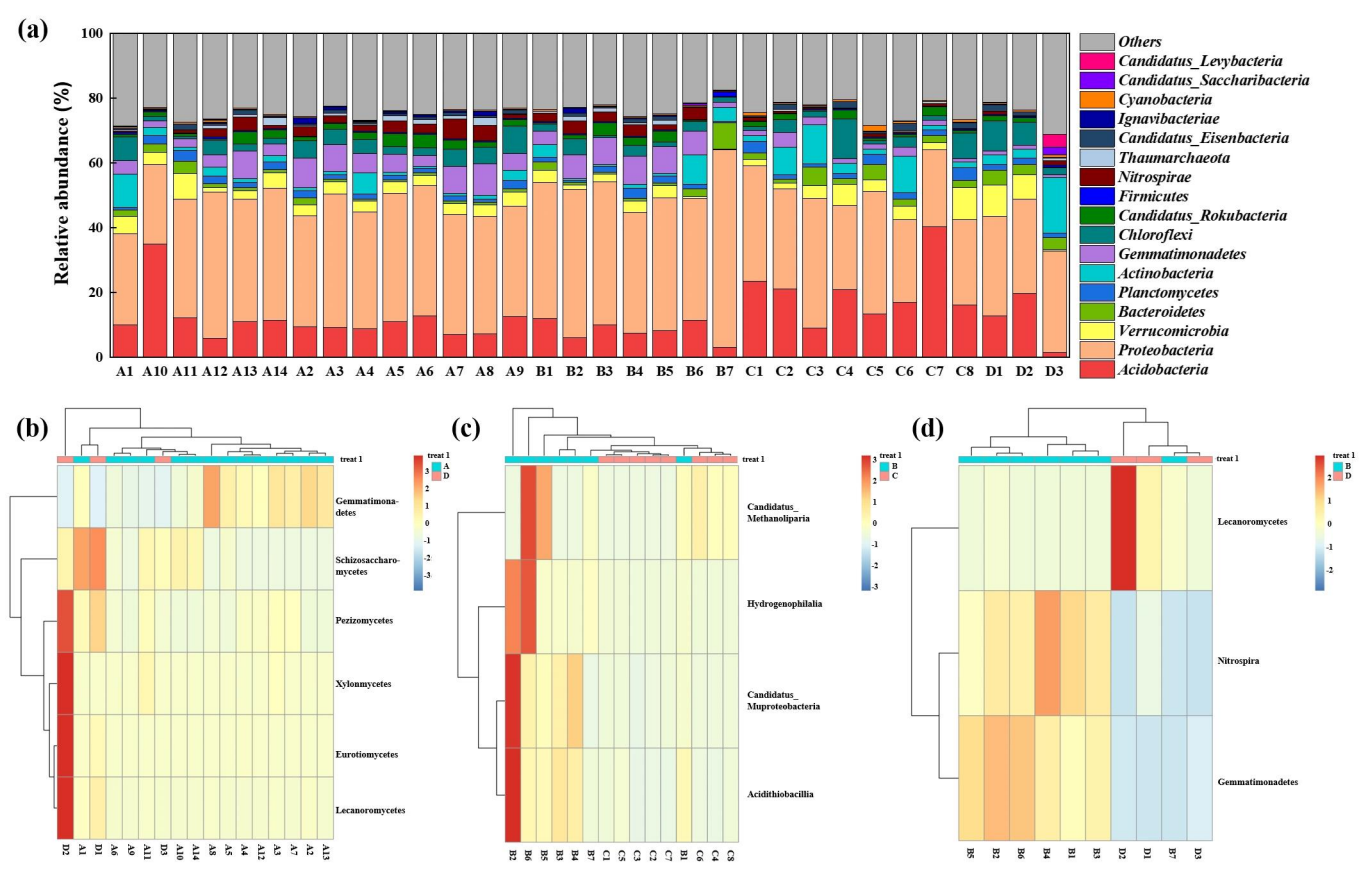
Microbial community composition and distinctive taxa across different functional zones of the Mn electrolysis plant. (**a**) Relative abundance of the dominant microbial phyla (average relative abundance >1%) across all soil samples. The “Others” category includes all phyla below 1% average abundance. (**b**–**d**) Heatmaps of the distinctive microbial classes in pairwise comparisons between different zones. Each panel represents a different comparison: (**b**) Zone A vs. Zone D, (**c**) Zone B vs. Zone C, and (**d**) Zone B vs. Zone D. Only taxa showing significant variation in the given comparison were displayed. Red indicates higher relative abundance.

The composition of the soil microbial community across different functional zones was illustrated in Figure 5a, which presented the community structure at the phylum and class levels for taxonomy with abundance exceeding 1%. The “Others” category incorporated fungal phyla (such as *Ascomycota* and *Basidiomycota)* along with other low-abundance bacterial and archaeal phyla. Overall, *Acidobacteria* and *Proteobacteria* emerged as the predominant microbial phyla across the manganese-contaminated site, highlighting their potential central role in this environment. Notable variations were observed in specific zones. The soil in the adjacent natural reservoir (Zone A) was characterized by a high abundance of microbial communities belonging to *Verrucomicrobia*, *Actinobacteria*, and *Gemmatimonadetes*. In the microbial communities surrounding the production and slag heap zones (Zone B), the phyla *Nitrospirae*, *Gemmatimonadetes*, and *Candidatus_Rokubacteria* demonstrated pronounced ecological advantages. Compared with Zones A and B, a higher abundance of *Acidobacteria*, a lower abundance of *Proteobacteria*, and a relatively high abundance of *Bacteroidetes* were found around the office zone (Zone C). The community composition of the sewage treatment unit (Zone D) was different from that of the other areas, with a higher abundance of *Actinobacteria* and *Chloroflexi*.

The differences in microbial community composition between different zones at the class level are shown in Figure 5b–d. Compared with Zone A, a rich fungal community, including *Lecanoromycetes*, *Eurotiomycetes*, *Pezizomycetes*, *Schizosaccharomyces*, and *Xylonomycetes*, was found in Zone D, which might be associated with the high levels of organic matter and Mn in the sewage treatment plant. Previous studies have indicated that fungi exhibit greater tolerance to Mn than bacteria because of their cellular structure and other inherent characteristics [20]. The primary distinction in the microbial communities between Zone B and Zone C was the higher abundance of *Hydrogenophilalia*, *Candidatus_Muproteobacteria*, *Candidatus_Methanoliparia*, and *Acidithiobacillia* in Zone B. These microorganisms have a high tolerance to manganese [21]. Compared with Zone D, the fungi *Lecanoromycetes,* the bacteria *Nitrospira,* and *Gemmatimonadetes* were more abundant than in Zone B, which was consistent with the results based on the phylum.

### 3.3. The Effect of Environmental Factors on Microbial Community

**Figure 6:**
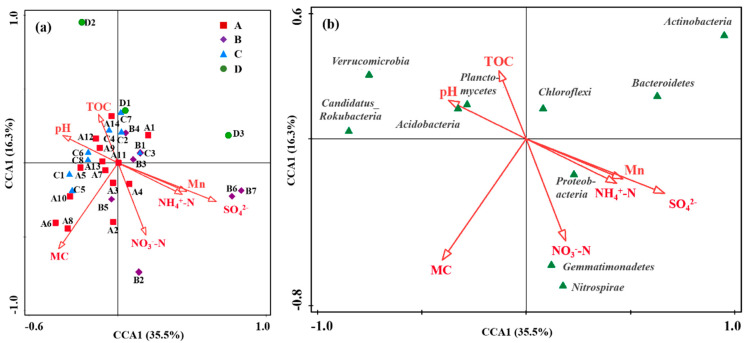
CCA analysis revealing the relationship between environmental factors and microbial community structure. (**a**) Distribution of sampling zones. (**b**) Distribution of major bacterial phyla. Arrows represent environmental variables (MC, moisture content; A, B, C, and D represent Zone A, B, C, and D).

The effect of various environmental factors on the microbial community structure was analyzed based on the physicochemical properties, Mn content, and microbial community composition in different zones (Figure 6). CCA analysis showed that environmental variables accounted for 35.5% of the total explanation of microbial communities, and samples from four different regions overlapped partially (Figure 6a). Specifically, samples from the production and slag heap zones and the office zone (Zones B and C) clustered closely along the positive direction of the vectors for Mn, SO_4_^2−^, and NH_4_^+^-N, indicating that their distinct microbial communities were primarily driven by the combined pressure of these industrial pollutants. Conversely, samples from the natural reservoir (Zone A) were positioned on the opposite side, correlating with NO_3_^−^-N and TOC, which was consistent with a less contaminated background. The dispersed distribution of samples from the sewage treatment unit (Zone D) suggested a more heterogeneous environment or influence from unmeasured variables.

The relationship between environmental characteristics (including moisture content, pH, TOC, NO_3_^−^-N, NH_4_^+^-N, SO_4_^2−^, and Mn) and the microbial community structure was also analyzed by CCA [13]. All environmental characteristics showed a significant influence on the bacterial community structure. The abundance of *Proteobacteria, Gemmatimonadetes,* and *Nitrosospira* was positively correlated with NO_3_^−^-N, NH_4_^+^-N, SO_4_^2−^, and Mn, but negatively with pH and TOC (Figure 6b). Conversely, the abundances of *Candidatus_Rokubacteria*, *Chloroflexi*, *Planctomycetes*, and *Acidobacteria* were positively correlated with pH and TOC. The positive correlation between *Acidobacteria* and pH might be attributed to the indirect effect of pH on manganese bioavailability. In this contaminated site, higher pH likely reduces soluble Mn toxicity, indirectly favoring the growth of *Acidobacteria* capable of tolerating the overall metal-stressed environment.

**Figure 7:**
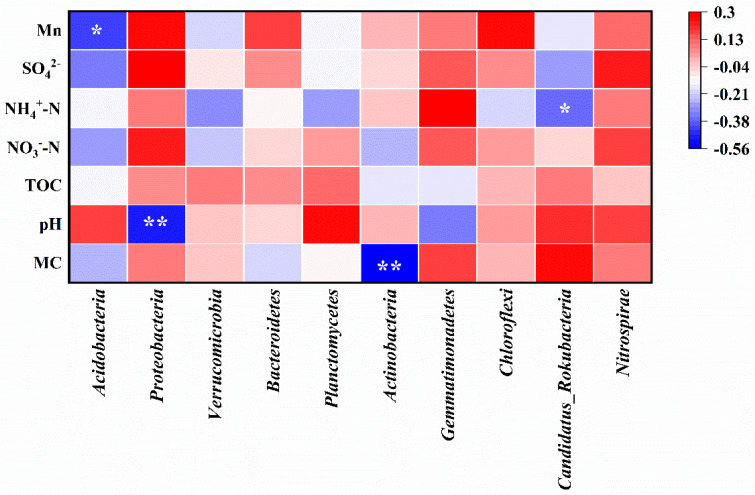
Spearman correlation analysis between environmental factors and soil microorganism communities at the phylum level (MC, moisture content; correlation coefficient r < 0 is the negative correlation, and r > 0 is positive correlation;* means *p* < 0.05, ** means *p* < 0.01).

Spearman correlation analysis between environmental factors and soil microorganism communities at the phylum level is shown in Figure 7. The abundance of *Acidobacteria* exhibited a significant negative correlation with Mn content (correlation coefficient r = −0.427, *p* < 0.05), as well as moisture content, NO_3_^−^-N, and SO_4_^2−^. The abundance of *Proteobacteria* was significantly negatively correlated with soil pH (r = −0.496, *p* < 0.01), while Mn, NO_3_^−^-N, and SO_4_^2−^ played positive roles in its abundance. The abundance of *Actinobacteria* showed a highly significant negative correlation with soil moisture content (r = −0.555, *p* < 0.01), while *Candidatus_Rokubacteria* exhibited a significant negative correlation with NH_4_^+^-N (r = −0.367, *p* < 0.05). Furthermore, the abundance of *Nitrospira* was positively correlated with all environmental factors in this study, suggesting that the soil in the Mn electrolysis plant area was conducive to its growth.

### 3.4. Distribution and Abundance of Manganese Resistance Genes

**Figure 8:**
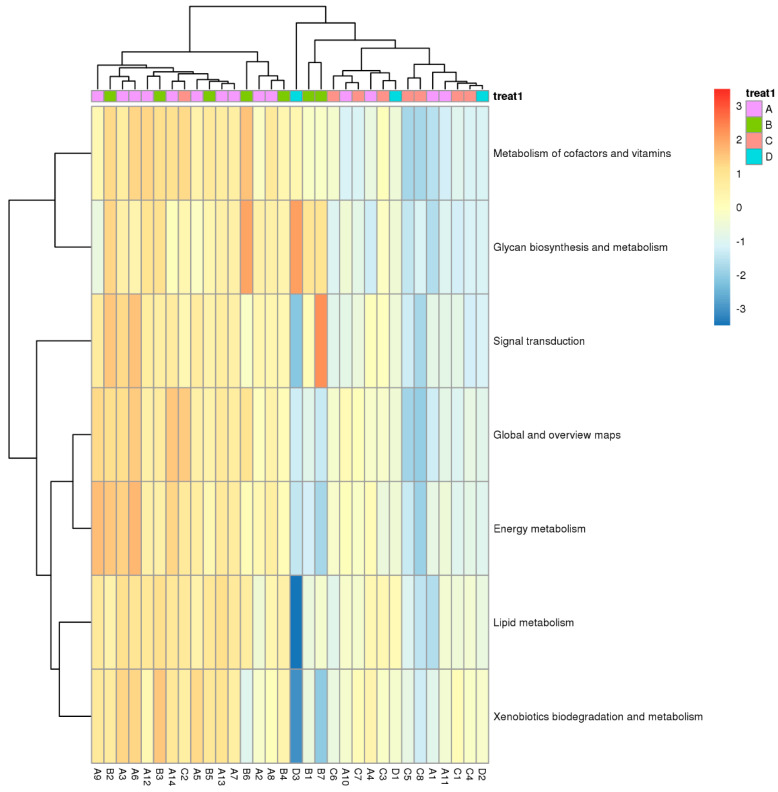
Heat map of relative abundance of differentially expressed genes based on KEGG database (ANOVA, *p* < 0.05; A, B, C, and D represent Zone A, B, C, and D.).

Functional differences of genes based on KEGG pathway analysis were performed using one-way ANOVA for different samples. The difference was mainly found in cofactor and vitamin metabolism and signal transduction. Furthermore, the heatmap reveals a concomitant upregulation in xenobiotic biodegradation and metabolism genes, indicating a potential exposure to other environmental pollutants. Genes in the surrounding area of the natural reservoir (Zone A) and the production and slag heap zones (Zone B) were very similar but significantly different from those in the office zone (Zone C) and sewage treatment unit (Zone D) (Figure 8).

**Figure 9:**
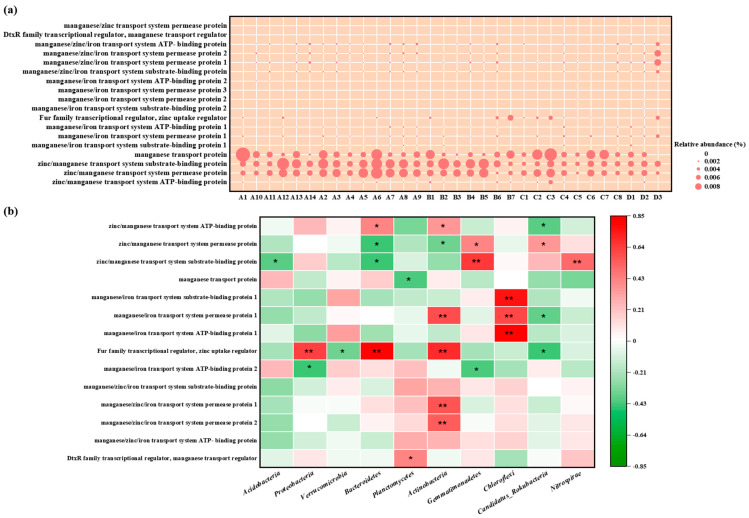
(**a**) Relative abundance of manganese resistance genes based on KEGG Database, (**b**) Spearman correlation analysis between manganese resistance genes and soil microorganism communities at the phylum level (A represents Zone A, which covers the adjacent natural reservoir; B, C, and D represent Zone B, Zone C, and Zone D, which are located at the production and slag heap zones, office zones, and sewage treatment units in the Mn electrolysis enterprise. Correlation coefficient r < 0 is a negative correlation, and r > 0 is a positive correlation. * means *p* < 0.05, ** means *p* < 0.01.).

The abundance of Mn-related genes among different regions and samples shown in Figure 9. Manganese transport protein, zinc/manganese transport system substrate-binding protein, and zinc/manganese transport system permease protein were highly abundant in various samples, which conferred Mn resistance to microorganisms [22]. There were some differences in the types and abundance of Mn resistance genes among the samples and regions, but these differences were not significant, which might be associated with the insignificant regional variance in Mn levels. For example, the Mn content in sample D3 amounted to 78,920 mg/kg, and the abundance of Mn resistance genes, including manganese/zinc/iron transport system permease protein 1 and manganese/zinc/iron transport system permease protein 2, was higher than that in other samples. The Mn content in sample A6 was 7680 mg/kg, while metagenomic analyses found that the abundance of genes encoding manganese transport protein, zinc/manganese transport system substrate-binding proteins, and zinc/manganese transport system permease proteins was extremely high, even higher than those in other samples with higher Mn levels. This may be attributed to the fact that these manganese resistance genes can only be induced by low Mn concentrations; others are stimulated by high Mn. This expression process induced by Mn is typically regulated and achieved by the DtxR family transcriptional regulator, manganese transport regulator MntR [23]. Among the different samples, manganese transporter protein transcriptional regulatory factors exhibited variance in abundance. These regulatory factors, stimulated by Mn, can either suppress or induce the expression of genes encoding manganese transporter proteins, and they are critical for microbial communities to tolerate Mn. However, no manganese oxidation-related genes, such as the multicopper oxidase gene *mnx*, have been identified [24], indicating that microorganisms in this mining area might not possess the ability to oxidize Mn.

To explore which microbial groups were most associated with manganese resistance, we correlated the abundance of key Mn-resistance genes with the relative abundance of the dominant bacterial phyla (Figure 9b). Overall, the relative abundance of Mn-resistance genes showed a significant positive correlation with most phyla, including *Proteobacteria*, *Actinobacteria*, and *Chloroflexi*, and a strong negative correlation with *Acidobacteria*. At the gene-specific level, the ubiquitous “Fur family transcriptional regulator” was positively correlated with *Proteobacteria*. This pattern suggested that Proteobacteria are the primary putative hosts and contributors to the Mn-resistance genetic pool in this environment.

## 4. Discussion

### 4.1. Elevated Mn Levels in Mining Area Soils

The physicochemical properties of the soils across the different functional zones show no significant difference, indicating the long-term, intensive industrial operations (e.g., dust deposition, leachate migration) have created a diffuse and widespread contamination, rather than confined pollution. This has led to a “homogenization” of the soil matrix across the site. However, the Mn level in different regions of this Mn electrolysis plant area far exceeds the background value (688 mg/kg) of soil Mn in Guizhou Province, with the highest reaching 78,920 mg/kg. More severe soil Mn pollution occurred in the production and slag heap zones (Zone B) and sewage treatment unit (Zone D). While the latest Risk Control Standard for Soil Contamination in China (GB 15618-2018 and GB 36600-2018) no longer lists Mn as a controlled pollutant [25,26], the data clearly demonstrated that the legacy of mining activity has resulted in severe Mn pollution. The fact that all sampling points exceeded the second-grade limit of 2000 mg/kg Mn for soils with a 6.5 < pH < 7.5 of the retired standard (GB 15618-1995) emphasized the persistent and widespread nature of the Mn contamination [27]. Excessive Mn can cause severe damage to human beings and the ecological environment [25]. Exposure to high concentrations of Mn can give rise to severe neurological disorders and “manganese pneumonia” [26]. In particular, if infants and young children are exposed to excessive Mn, it may have long-term implications for the nervous system [18]. Such extreme metal loading in this Mn electrolysis plant area can act as a powerful selective pressure, inevitably driving the restructuring of soil microbial communities [27].

### 4.2. The Dominant Bacterial Phyla Acidobacteria and Proteobacteria

*Acidobacteria* and *Proteobacteria* are the dominant bacterial phyla across the entire mining area. Similarly, previous studies demonstrated that *Proteobacteria*, *Actinobacteria*, and *Acidobacteria* were the predominant bacteria in manganese mining areas [28]. *Acidobacteria* can tolerate certain concentrations of Mn and oxidize or adsorb Mn(II) to efficiently remove Mn [29]. *Actinobacteria* exhibit resistance to multiple heavy metals, including Zn, Cu, and Mn [30]. Mn can promote the activity of *Actinobacteria* and increase their abundance in the soil [31]. The soil microorganisms in the abandoned Mn-contaminated mine tailings were predominantly *Proteobacteria*, accounting for more than 85% of the total microbial community [32]. Bacteria within the phylum *Proteobacteria*, such as the Mn(II)-oxidizing bacterium *Pseudomonas aeruginosa*, have been shown to possess a certain level of Mn tolerance [33]. The average Mn concentration in Zone B reached up to 33,304 mg/kg, resulting in a relatively simple microbial community. And certain specific groups of microorganisms with Mn resistance exhibited high abundance. Mn accumulation in soil can provide Mn-tolerant bacteria with advantages in thriving in their niches [34].

The absence of significant differences in α-diversity, coupled with the profound shifts in community composition (β-diversity), suggested the strong and pervasive selective pressure of Mn contamination based on the ecological concept of functional redundancy [35]. Under severe Mn contamination, the soil environment acts as a filter, eliminating sensitive taxa regardless of their original functional zone. This leads to a convergence in the overall number of species (α-diversity) but a drastic turnover in the identity of those species (β-diversity). The significant PERMANOVA result confirmed that Mn contamination was a key driver of this community turnover. The resulting communities, though taxonomically distinct, were all dominated by Mn-tolerant lineages, primarily from the phyla *Acidobacteria* and *Proteobacteria.*

Furthermore, the significant positive correlation between the abundance of *Proteobacteria* and the total load of Mn-resistance genes (Figure 9b) indicated that this phylum was taxonomically dominant but also functionally critical for manganese adaptation. The negative correlation with *Acidobacteria* suggested a different survival strategy or sensitivity to the specific Mn stress regime. This gene-taxa correlation directly links the shift in community structure (dominated by *Proteobacteria*) to the enrichment of a specific function (Mn resistance), providing a more solid understanding of adaptation beyond taxonomic profiling alone. Therefore, key phyla, like *Acidobacteria* and *Proteobacteria*, are not merely present but active in metal tolerance and biogeochemical cycling, particularly of Mn.

The prevalence of metal-tolerant taxa, especially Mn-resistant bacteria within the *Proteobacteria* and *Acidobacteria*, points to the innate potential for natural attenuation. The microbial community is already performing essential ecosystem services by immobilizing metals and cycling nutrients. This presents an opportunity for targeted bioremediation strategies, such as bioaugmentation with native, high-performing strains like *Pseudomonas aeruginosa*, or biostimulation to enhance the activity of indigenous metal-oxidizing communities.

### 4.3. The Potential Adaptive Mechanism of Soil Microbial Communities

CCA and Spearman’s rank correlation analysis suggested that several environmental parameters, including Mn content, NO_3_^−^-N, NH_4_^+^-N, pH, and moisture, showed an influence on the microbial community structure, among which Mn content was the primary factor. High Mn levels in this Mn electrolysis plant area increased the abundance of *Proteobacteria*, *Acidobacteria*, *Bacteroidetes*, *Chloroflexi*, and *Nitrospira*. These findings align with previous research [11]. It indicated that Mn contamination acted as a critical environmental filter, shaping a specialized microbial community in the mining area. The observed community shift suggested a selection for organisms possessing adaptive traits for Mn resistance. The prominence of nitrogen-cycling bacteria, such as *Nitrospira*, is particularly noteworthy. It pointed to a potential interaction between Mn biogeochemistry and the nitrogen cycle through the metabolic activity of microbes that can utilize Mn redox cycling for energy or detoxification [36]. Consequently, the microbial community adapted to the harsh conditions not only by selecting for general Mn-resistant populations but also by potentially boosting specific biogeochemical processes. This established a resilient ecosystem where Mn-resistant bacteria formed the core functional community, playing a pivotal role in the biogeochemical cycling of metals and nutrients within this contaminated environment.

The ultimate evidence of Mn’s impact lay not in taxonomy but in function. The metagenomic analysis revealed that the compositional shifts observed in Figure 5 were directly linked to the enrichment of Mn-resistance genes in the most contaminated zones. Thus, the impact of Mn is most accurately measured by its role in selecting for an “Mn-adapted microbiome,” characterized by a distinct community structure and an enriched arsenal of resistance mechanisms, rather than by a simple reduction in species counts. The abundance and diversity of manganese resistance genes (including manganese transporters, manganese transporter transcriptional regulators, etc.) further corroborated the dominant position of Mn-resistant bacteria within the soil microbial community. These genes encode key components of the microbial Mn resistance mechanism. Manganese transport proteins can recognize and translocate Mn(II) and then absorb or expel it across the membrane to maintain Mn homeostasis, thereby preventing cell damage [37]. Transcriptional regulators of manganese transporter proteins are responsible for regulating the expression of manganese transporter proteins in response to Mn, which plays a key role in maintaining Mn homeostasis [38]. The pervasive distribution of Mn resistance genes is not only a microbial response but also evidence of a powerful and persistent selection pressure exerted by Mn contamination. These genes, essential for cellular detoxification and homeostasis, provided the biochemical foundation that allows soil microbes to survive. Their abundance across the population buffered the entire microbial community against Mn toxicity, maintaining metabolic activity and ensuring the continuity of critical soil processes, such as the previously mentioned nitrogen cycling.

In summary, the adaptive microbial community in this Mn electrolysis plant exemplifies a powerful environmental selection process driven by metal stress. Soil Mn level acts as a stringent filter, directly driving a phylogenetic shift towards the dominance of *Proteobacteria* and *Acidobacteria*. This community restructuring is underpinned by pervasive genetic adaptation, notably the widespread abundance and diversity of Mn resistance genes. Mn exposure triggers the overexpression of these genes, enabling resistant microbes, particularly within these dominant phyla, to thrive by regulating intracellular homeostasis, while non-resistant populations are progressively eliminated. Consequently, the community is structured through a selection process that enriches tolerant taxa, shapes a shared genetic resistance reservoir, and assembles a resilient, functionally adapted ecosystem. This highlights how severe contamination disrupts natural random assembly, leading to a predictable but low-diversity state that disrupts ecological balance. While adapted, such innate communities are insufficient for remediation. These findings advocate for re-evaluating manganese in environmental risk assessments and underscore the need for targeted strategies to mitigate threats to the ecosystem and public health.

## Data Availability

The original contributions presented in this study are included in the article. Further inquiries can be directed to the corresponding author.
